# Outcomes of prophylactic lauromacrogol injection versus non-injection in patients with endogenous cesarean scar pregnancy treated by hysteroscopic surgery: a retrospective cohort study

**DOI:** 10.1186/s12884-023-06088-1

**Published:** 2023-11-04

**Authors:** Lei Lu, Yiming Shao, Zhaoyang Qu, Guilian Huang, Suping Lang, Caiqun Yang, Siqi Lang, Shuying Fang

**Affiliations:** 1 Department of Gynecology, Hangzhou Fuyang Women and Children Hospital, Hangzhou, China; 2 Department of Ultrasonography, Hangzhou Fuyang Women and Children Hospital, Hangzhou, China; 3 Medical Record Room, Hangzhou Fuyang Women and Children Hospital, Hangzhou, China

**Keywords:** Hysteroscopy, Cesarean scar pregnancy, Cesarean section, Lauromacrogol

## Abstract

**Background:**

This study aimed to investigate the efficacy of hysteroscopic surgery for endogenous cesarean scar pregnancy (CSP) and the value of prophylactic ultrasound-guided local injection of lauromacrogol.

**Methods:**

This retrospective study included 131 patients diagnosed with endogenous CSP who underwent hysteroscopic surgery at the Hangzhou Fuyang Women and Children Hospital between January 2018 and May 2022. Lauromacrogol (10–20 mL) was administered within 24 h preoperatively using an ultrasound-guided vaginal injection to 78 patients (L group) versus not administered to 53 patients (non-L group). Their clinical data and outcomes were analyzed.

**Results:**

Mean gestational age, gestational mass size, and uterine scar thickness and median preoperative blood β-human chorionic gonadotropin levels of the non-L versus L groups were 46.26 versus 45.01 days, 2.05 versus 2.39 cm, 0.35 versus 0.32 cm, and 19850.0 versus 26790.0 U/L, respectively (*P* > 0.05 for each). The non-L and L groups had similar success rates (98.1% vs. 98.7%, *P* = 1.0). Complications related to lauromacrogol administration, including abdominal pain, massive bleeding, and bradycardia, were experienced by 46.2% (36/78; *P* < 0.001) of L group patients. The non-L had a significantly shorter mean hospital stay (4.85 ± 1.12 vs 5.44 ± 1.08 days) and lower total cost (6148.75 ± 1028.71 vs 9016.61 ± 1181.19) (*P* < 0.01).

**Conclusions:**

Hysteroscopic surgery is effective and safe for patients with endogenous CSP. Prophylactic lauromacrogol injection increases the incidence of complications and costs. Direct hysteroscopic surgery can reduce pain and financial burden in patients with endogenous CSP and save medical resources for other patients.

## Background

Cesarean scar pregnancy (CSP) is a rare condition involving gestational sac implantation in a uterine incision scar [1]. The rates of cesarean sections and ultrasound diagnoses are increasing annually [2]. In 2000, Vail [3] classified CSP into two types: type 1, also known as the endogenous type, in which gestational sacs are implanted shallowly at the scar and mostly grow toward the uterine cavity; and type 2, also known as the exogenous type, in which the implantation occurs more deeply at the scar defect and bulges toward the bladder. CSP may involve life-threatening complications, such as massive vaginal bleeding and uterine rupture, because of the thin muscle wall of the scar, which reduces uterine contractility.

Consensus is currently lacking on a standardized therapy for CSP in clinical practice [4]. Common treatments include medical therapy, ultrasound-guided curettage, uterine artery embolization (UAE), and curettage after hysteroscopy. Effective treatment should both prevent the occurrence of severe blood loss and preserve fertility, women’s health, and quality of life [5, 6].

Blocking blood flow to the sac using UAE results in embryo death and reduces bleeding during curettage. UAE followed by curettage has been used in the treatment of CSP because of its minimal invasiveness and efficiency; however, it may cause fever, pelvic pain, post-embolization syndrome, and pulmonary embolism [6, 7].

Lauromacrogol (polidocanol; polyoxyethylene [8] lauryl ether) is the most commonly used foam sclerosant [9]. It has been used in sclerotherapy for CSP via local injection to block the veins around the gestational sac located at the cesarean scar, kill the embryo, and prevent massive bleeding during curettage. Instead of UAE, it has been widely applied in CSP before hysteroscopic surgery [10] or curettage [8]. Compared with UAE, the local injection of lauromacrogol causes less damage, costs less, and involves fewer complications; however, controlled studies of its non-use are lacking. Here we introduce a treatment for CSP and investigate the cost and efficacy of ultrasound-guided local lauromacrogol injection combined with curettage and hysteroscopy compared with no pretreatment.

## Methods

### Study patients

We enrolled 148 patients diagnosed with CSP at the Department of Gynecology of Hangzhou Fuyang Women and Children Hospital from January 2018 to May 2022. The patients’ clinical and anamnestic data were extracted from their medical records and short-term follow-ups, and 131 patients treated with hysteroscopy were included in the analysis. Depending on whether preprocessing was performed by the local injection of lauromacrogol, the participants were divided into L and non-L groups.

### Diagnosis

The diagnosis of CSP was made considering a history of cesarean delivery and serum β-human chorionic gonadotropin (β-hCG) and transvaginal ultrasonographic findings that meet the following criteria [2]: 1) empty cervical canal and uterine cavity without sac contact; 2) gestational sac at the anterior wall of the isthmic segment with or without cardiac activity; 3) an obvious myometrial defect between the sac and the bladder; and 4) functional placental/trophoblastic circulation surrounding the gestation sac/mass. According to the Chinese Medical Association [11], patients with a > 3-mm-thick residual muscle layer in the uterine scar were classified as having CSP type 1, while those with a ≤ 3-mm-thick but > 1-mm-thick residual muscle layer were classified as having CSP type 2. Both types are approximately equivalent to the type 1 Vail standard, that is, the endogenous type.

### Ultrasound-guided local lauromacrogol injection

All patients underwent routine preoperative examinations, and patients in the L group underwent ultrasound-guided lauromacrogol injection. The patients emptied their bladders and were placed in a lithotomy position. The physician disinfected the perineum and vagina and covered them with sterile towels. Two milliliters of sulfur hexafluoride microbubbles (SonoVue, Bracco Suisse, SA) was injected as a contrast agent through the antecubital vein. The patient underwent transvaginal ultrasound (Philips EPIQ5G ultrasonic system) for the observation of blood flow around the gestational sac and guidance of the puncture. A 21-gauge needle (Hakko, Tokyo, Japan) was used to inject 10–20 mL of lauromacrogol into the anterior isthmus muscle layer around the gestational sac at multiple points, and the gestational sac was flaky or annular-enhanced. The peripheral blood flow was sparse and eventually stopped.

### Hysteroscopy

The hysteroscopic surgery was performed by qualified senior doctors using abdominal ultrasound monitoring. In the L group, hysteroscopic surgery was performed within 24 h of the local injection. All patients were prepared for intrauterine balloon or gauze tamponade and transvaginal or laparoscopic and open surgery and were informed of the surgical risks and options. Under intravenous or epidural anesthesia, the cervix was dilated and the uterine cavity, cervical canal, and pregnancy attachment site were checked under hysteroscopic guidance. After most of the decidua and pregnancy were removed using an electric suction device under abdominal ultrasound guidance, the hysteroscopy was performed to check for any residue or implantation. In cases of bleeding, visible villi were removed. The specimens were sent for pathological examination.

### Observation indicators

The gestational age, thickness of the muscle layer at the scar, length of the gestational mass, and preoperative blood hCG levels were compared between groups, and bleeding volume, intraoperative findings, and use of uterine tamponade were recorded. The amount of bleeding was calculated intraoperatively using an estimation method and postoperatively using a weighing method. On the first postoperative day, routine blood examinations were repeated to understand the change in hemoglobin content; serum β-hCG was rechecked on the first and third postoperative days. Patients who demonstrated satisfactory decreases in blood hCG levels and reduced vaginal bleeding were discharged from the hospital.

The patients were instructed to submit samples for weekly blood hCG level testing as outpatients until the results were normal. B-ultrasound was reviewed within 1 month postoperative to observe abnormal mass formation in the anterior isthmus and uterine cavity. If the blood hCG level decreased to normal and the anterior isthmus mass disappeared, the initial treatment was considered successful. The hysteroscopic surgery was considered a failure if the blood hCG level decreased by < 50% weekly after the operation or if the mass in the anterior isthmus of the uterus persisted and a second operation was required. The length of hospital stay, expenses, and occurrence of complications, such as abdominal pain, major bleeding, nausea, and vomiting, were recorded. The primary outcome measure was the cure rate after hysteroscopic surgery defined as the percentage of patients who were successfully treated with hysteroscopic procedures compared to the total number of patients treated. Failure was defined as the requirement for a second surgery or transition to another surgical method. The secondary outcomes were hospital stay duration, hospital stay cost, and incidence of complications related to the lauromacrogol injection.

### Statistical analysis

A descriptive analysis was performed of all patients’ data. Categorical variables are reported as absolute numbers and proportions (%). Continuous data are expressed as mean and standard deviation or median and interquartile range according to the presence or absence of a normal distribution. Variables were compared using the chi-squared test (categorical variables), one-way analysis of variance (normal distribution), and Kruskal–Wallis (skewed distribution) tests. For categorical data, Fisher’s exact test was applied if the n was < 5. All analyses were performed using the statistical software package R 3.3.2 (http://www.R-project.org; The R Foundation) and Free Statistics software version 1.61. A two-tailed test was performed, and values of *P* < 0.05 was considered statistically significant.

## Results

### Characteristics of study population

**Fig. 1:**
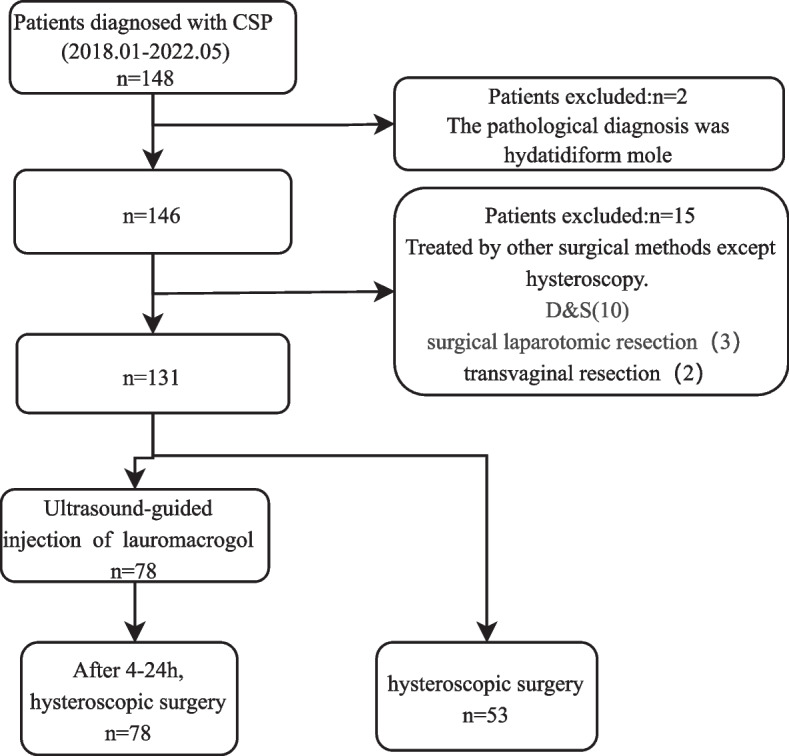
Flowchart of participant selection process. CSP, cesarean scar pregnancy; D&S, dilation and manual and vacuum aspiration

This study included 148 patients diagnosed with CSP at the Department of Gynecology of Hangzhou Fuyang Women and Children Hospital between January 2018 and May 2022. After excluding patients with hemodynamic instability due to massive vaginal bleeding, primary surgery, or surgical methods other than hysteroscopy, 131 participants (78 in L group, 53 in non-L group) were included in the analyses (Fig. 1).

All patients had a previous history of cesarean delivery. No statistically significant intergroup differences were found in age, fetal heart activity rate, gestational age, interval from recent caesarean section, serum β-hCG level, myometrial thickness, and pretreatment sac diameter. The participants’ demographic and clinical characteristics are summarized in Table 1.

**Table 1:** Participants’ general clinical characteristics by study group

Parameters	Non-L group (*n* = 53)	L group (*n* = 78)	*P* value
Age (years)	34.64 ± 4.82	34.13 ± 5.05	0.562
Gestational age (days)	46.26 ± 6.99	45.01 ± 9.07	0.399
Fetal heartbeat positive, n (%)	21 (39.6)	26 (33.3)	0.461
Gravidity (n)	4.0 (3.0, 5.0)	4.0 (3.0, 5.0)	0.160
Abortion (n)	2.0 (1.0, 3.0)	2.0 (1.0, 3.0)	0.677
Number of cesarean deliveries			0.144
1, n (%)	23 (43.4)	44 (56.4)	
≥ 2, n (%)	30 (56.6)	34 (43.6)	
Time from cesarean delivery (years)	5.0 (3.0, 9.0)	5.0 (2.0, 8.0)	0.285
Type of CSP, n (%)			0.651
Type 1	36 (67.9)	50 (64.1)	
Type 2	17 (32.1)	28 (35.9)	
Preoperative serum hCG level (IU/L)	19850.0 (7366.0, 48560.0)	26790.0 (10170.0, 55730.0)	0.137
Uterine scar thickness (cm)	0.35 ± 0.16	0.32 ± 0.12	0.121
Mass size (cm)	2.05 ± 0.92	2.39 ± 1.31	0.109

Data are presented as mean ± standard deviation, median (25^th^, 75^th^ percentile), or n (%). Values of *P* < 0.05 were considered statistically significant

### Outcomes of women with CSP treated with versus without lauromacrogol injection prior to hysteroscopy

None of the patients were lost to follow-up. No significant intergroup differences were noted in time to β-hCG normalization and bleeding stoppage during treatment and follow-up (Table 2).

**Table 2:** Comparison of surgical conditions and clinical outcomes between the two groups

Parameter	Non-L group (*n* = 53)	L group (*n* = 78)	*P* value
Cure rate (%)	52 (98.1)	77 (98.7)	1
Operating time (min)	29.87 ± 13.51	29.58 ± 19.99	0.926
Intraoperative blood loss (mL)	15.0 (10.0, 20.0)	20.0 (10.0, 30.0)	0.105
Hemoglobin change	12.0 (4.0, 16.0)	10.5 (6.0, 17.0)	0.952
Time to β-hCG normalization (days)	28.07 ± 8.51	29.39 ± 9.92	0.586
Complications (%)			< 0.001*
Overall	0 (0)	36 (46.2)	
Abdominal pain	0 (0)	28 (35.9)	
Massive bleeding	0 (0)	5 (6.4)	
Bradycardia	0 (0)	3 (3.8)	
Uterine packing rate (%)	2 (3.8)	7 (9.0)	0.311
Hospital day(day)	4.85 ± 1.12	5.44 ± 1.08	0.003*
Hospital stay cost (¥)	6148.75 ± 1028.71	9016.61 ± 1181.19	< 0.001*
Surgery	1266.12 ± 282.97	1296.41 ± 265.14	0.535
Medical supplies	155.9 (21.0, 819.0)	1348.0 (1062.0, 1548.0)	< 0.001*
Medication	508.03 ± 225.35	2033.06 ± 431.73	< 0.001*
Ultrasound and imaging	495.36 ± 140.62	709.52 ± 137.57	< 0.001*

Data are presented as mean ± standard deviation, median (25^th^, 75^th^ percentile), or n (%). *Statistically significant

Success rates were similar in the two groups. One patient from each group experienced failure of hysteroscopic surgery. In the non-L group, the thickness of the lower uterine segment in the failed case was 1.3 mm, long diameter of the gestational mass was 2.8 cm, and intraoperative bleeding was approximately 120 mL. After postoperative monitoring, the serum hCG level decreased slowly and remained at 3455 IU/L at 26 days postoperative. Ultrasonography suggested that the anterior isthmus mass had an enriched blood flow signal that was slightly convex to the bladder. Treatment failure was resolved with transvaginal residual embryo removal and scar repair. In the L group, the thickness of the lower uterine segment in the failed case was 2.5 mm and long diameter of the gestational mass was 6.0 cm. Owing to the large amount of bleeding during hysteroscopic surgery, surgeons switched to laparoscopic surgery in emergencies.

In the L group, 36 patients presented with complications, including 28 with abdominal pain, five with massive bleeding (of whom two required emergency surgery), and three had bradycardia with a heart rate < 50 beats per minute accompanied by nausea and vomiting in one. Significant differences were noted in cost and length of hospital stay (Table 2).

## Discussion

### Advantages of hysteroscopic surgery over curettage in treating CSP

The special implantation position of the CSP creates a risk of uterine rupture and massive hemorrhage as pregnancy progresses. After a clear diagnosis is made, early termination is recommended, and surgery is the first choice. In a multicenter retrospective study, for cases of endogenous CSP in which the myometrial thickness was > 3 mm, the curettage group showed the shortest and lowest expenses, but the hysteroscopy group had the highest success rate (95.9% vs the 84.0% success rate of the curettage group) [12]. Through this retrospective analysis of 131 patients diagnosed with CSP who were treated with hysteroscopy, we found that hysteroscopy is an efficient, safe, and minimally invasive treatment for CSP. Qiu et al. [13] also found that, compared to ultrasonography-guided dilation and curettage, patients with CSP treated with hysteroscopy had significantly lower rates of hospitalization duration, intraoperative blood loss, and overall complications. Hysteroscopic surgery can also be selected for patients with failed curettage, with a success rate as high as 95.6% [14]. This indicates that hysteroscopy is superior to curettage and may be the first-line method for CSP. Subgroup analysis of all outcomes according to CSP classification showed that hysteroscopic surgery was equally effective in both type 1 and type 2 CSP patients (Tables 3 and 4).

**Table 3:** Comparison of general clinical characteristics between type 1 and type 2

Parameters	Type 1(*n* = 86)	Type 2 (*n* = 45)	*P*
Age (years)	34.1 ± 4.9	34.8 ± 5.0	0.397
Gravidity (n)	4.0 (3.0, 5.0)	4.0 (3.0, 5.0)	0.289
Abortion (n)	2.0 (1.0, 3.0)	2.0 (1.0, 3.0)	0.486
Number of cesarean deliveries (*n*)			0.717
1, n (%)	43 (50)	24 (53.3)	
≥ 2, n (%)	43 (50)	21 (46.7)	
Fetal heartbeat positive, n (%)	28 (32.6)	19 (42.2)	0.273
Time interval from cesarean delivery (years)	5.0 (2.0, 8.0)	6.0 (3.0, 9.0)	0.232
Gestational age (days)	45.5 ± 8.6	45.7 ± 7.8	0.894
Size of mass (cm)	2.1 ± 1.0	2.6 ± 1.4	0.009
Pre-operative serum hCG level (IU/L)	19450.0 (8572.0, 39900.0)	33640.0 (12810.0, 66650.0)	0.015

**Table 4:** Comparison of surgical conditions and clinical outcomes between type 1 and type 2

Parameters	Type 1(*n* = 86)	Type 2 (*n* = 45)	*P*
Treated by Lauromacrogol(%)			0.651
No	36 (41.9)	17 (37.8)	
Yes	50 (58.1)	28 (62.2)	
Hemoglobin change(g/L)	11.0 (4.0, 18.0)	11.0 (7.0, 16.0)	0.737
Hospital day	5.1 ± 1.1	5.3 ± 1.1	0.323
Hospital stay cost (¥)	7764.1 ± 1767.3	8010.2 ± 1880.2	0.464
Intraoperative blood loss (ml)	17.5 (10.0, 20.0)	20.0 (10.0, 40.0)	0.138
Operating time (min)	28.8 ± 12.2	31.5 ± 25.0	0.407
Time to β-hCG normalization (days)	28.7 ± 9.0	28.6 ± 9.7	0.973
Uterine packing rate (%)	5 (5.8)	4 (8.9)	0.493
Complications (%)	20 (23.3)	16 (35.6)	0.134
Cure rate (%)	86 (100)	43 (95.6)	0.116

### Value of lauromacrogol pretreatment

Ultrasound-guided local lauromacrogol injections combined with curettage or hysteroscopy have been widely used to treat CSP in many hospitals, particularly in China [15, 16]. This retrospective study compared the efficacy of lauromacrogol injections administered to patients with CSP prior to hysteroscopic surgery and evaluated the value of lauromacrogol pretreatment in endogenous CSP. No obvious intergroup difference was noted in intraoperative blood loss or initial success rate. Thus, the pretreatment injection of lauromacrogol did not result in better outcomes.

Why does this seem contradictory to common sense? Lauromacrogol is widely used in sclerotherapy. First, its injection near a vein may cause venous fibrosis around the location, leading to vascular compression and hemostasis. Furthermore, the intravascular injection of lauromacrogol can harm endothelial cells in target vessels, thereby promoting local thrombosis [17, 18], eventually reducing bleeding. The rationale for reduced bleeding is that the local injection of lauromacrogol might block venous blood flow. Chen et al. [19] reported that the value of prophylactic UAE during hysteroscopy in patients with type II CSP was uncertain. Wang et al. [20] found that, compared with UAE pretreatment in the treatment of CSP, the pretreatment with local pituitrin injection appears to be the same: effective, more economical, and with fewer side effects.

In summary, we can conclude that, for major bleeding in patients with CSP, the most effective and commonly used hemostasis measure mainly promotes uterine smooth muscle contraction, which can stress the vessels in the uterus and reduce blood loss. Blocking the arterial or venous vessels cannot reduce massive hemorrhage. This may explain why the L group did not have an obvious advantage over the non-L group in reducing bleeding for CSP in hysteroscopy.

In this study, some patients experienced abdominal pain, vaginal bleeding, bradycardia, and other complications after the injection of lauromacrogol, which increased pain and fear in patients and was considered related to ischemic stimulation of the implantation site and premature stripping of the fetal membranes. Hospital costs and time were significantly higher in the lauromacrogol versus non-lauromacrogol group, suggesting that lauromacrogol increased the medical expenses and prolonged the hospital stay.

### Limitations

This study had several limitations. First, as the study was retrospective, detailed information was not always available. The degree of blood loss was visually assessed as well as weighed when possible by gynecologists; thus, it was not as accurate as expected. Second, the experience and skills of the different gynecologists may have influenced our conclusions owing to the lack of standard surgical procedures for CSP, which may have more promotional value in different hospitals. Third, most cases occur at < 8 weeks’ gestation, and the long diameter of the gestational sac is < 3 cm. Thus, the effects of hysteroscopic surgery in high-risk patients with CSP require further investigation.

## Conclusions

Hysteroscopic surgery is effective and safe in patients with endogenous CSP. However, the value of the local prophylactic injection of lauromacrogol remains uncertain. Pretreatment with lauromacrogol did not improve the patients’ clinical outcomes; rather, it increases the incidence of complications as well as the medical costs and length of hospital stay. Direct hysteroscopic surgery can reduce patient pain and economic burden as well as conserve medical resources.

## Data Availability

All data generated or analyzed during this study are included in this published article.

## Abbreviations

β-hCG: Beta human chorionic gonadotropin
CSP: Cesarean scar pregnancy
UAE: Uterine artery embolization
